# Antibacterial and Cytotoxic Activities of *Pinus tropicalis* and *Pinus elliottii* Resins and of the Diterpene Dehydroabietic Acid Against Bacteria That Cause Dental Caries

**DOI:** 10.3389/fmicb.2019.00987

**Published:** 2019-05-07

**Authors:** Kelly Regina da Silva, Jaqueline Lopes Damasceno, Moana de Oliveira Inácio, Fariza Abrão, Natália Helen Ferreira, Denise Crispim Tavares, Sergio Ricardo Ambrosio, Rodrigo Cassio Sola Veneziani, Carlos Henrique Gomes Martins

**Affiliations:** ^1^Laboratory of Research in Applied Microbiology (LAPEMA), University of Franca, Franca, Brazil; ^2^Institute of Biomedical Sciences (ICBIM), Federal University of Uberlândia, Uberlândia, Brazil

**Keywords:** dental caries, cariogenic bacteria, antibacterial activity, cytotoxic activity, *Pinus*

## Abstract

Considering the impact of dental caries on public health and the wide biological potential described for species belonging to the genus *Pinus*, here we investigate the antibacterial activity of the *P. elliottii* and *P. tropicalis* resins and of the diterpene dehydroabietic acid (DHA) against cariogenic bacteria. For this purpose, we have determined the minimum inhibitory concentration (MIC) and the minimum bactericidal concentration (MBC) of the resins and of the diterpene. We have also investigated the biofilm inhibition ability (through MBIC_50_ determination), as well as the synergistic effect (through fractional inhibitory concentration index) and the cytotoxic potential (through XTT assay) of the *P. elliottii* and *P. tropicalis* resins. The resins gave promising MIC and MBC values, which ranged from 12.5 to 400 μg/mL; DHA provided MIC and MBC values ranging from 25 to 400 μg/mL. The MICB_50_ values ranged from 0.78 to 400 μg/mL for the resins. Neither additive nor synergistic effects emerged for the combinations of one of the resins with chlorhexidine. The cytotoxic activity was ≥312.5 and ≥156.3 μg/mL for the *P. elliottii* and *P. tropicalis* resins, respectively. The resins showed antibacterial activity against planktonic and sessile cariogenic bacteria. These data are relevant and encourage further research into these plants, which may contribute to the discovery of new substances that can inhibit the growth of cariogenic microorganisms and reduce the incidence of dental caries.

## Introduction

The oral cavity is colonized by a group of intricate microorganisms that grow as a distinct oral biofilm, the dental plaque. This tooth-adhering film causes the most common oral microbial infection known to man, the so-called dental caries (Sunitha et al., 2017).

Dental caries is the most prevalent chronic disease worldwide and represents a costly burden to health care services especially in developing countries, where investments in prevention are minimal, and access to care is limited. Dental caries is a multifactorial disease that affects approximately 90% of the world population, with biological, environmental, and socio-behavioral risk factors contributing to its initiation and progression (Elamin et al., 2018). Microorganisms like *Streptococcus mutans* initiate the carious lesion on the tooth. *Streptococcus sanguinis*, *Streptococcus salivarius*, *Streptococcus mitis*, *Streptococcus sobrinus*, and *Lactobacillus* spp., among others, are the chief cariogenic microorganisms (Sunitha et al., 2017).

Given the current worldwide overuse of antibiotics in dentistry and the limited awareness of infection prevention guidelines, drug-resistant bacteria have become further hazards (Barenghi et al., 2018). Besides that, oral diseases can be so painful that they can obstruct food mastication and ingestion, thereby impairing the body nutritional status and immune function and negatively impacting the quality of life (Chinsembu, 2016; BaniHani et al., 2018). Against this backdrop, there is an urgent need to accelerate bioprospecting efforts and to isolate active compounds that may be used to manufacture new drugs and hygiene products for oral health.

In this context, plants stand out for having proven to be a promising source for the development of medicines to treat a broad spectrum of diseases (Cragg et al., 2014; Newman and Cragg, 2016). Plant oleoresins, which are substances containing resins in their composition (non-volatile principles), have raised interest due to their already reported antimicrobial potential (Caetano da Silva et al., 2014; Leandro et al., 2014; Fernández et al., 2018; Arruda et al., 2019).

The genus *Pinus* belongs to the family Pinaceae, includes many species, mainly trees, and is found mostly in the northern hemisphere. One of the features of *Pinus* species is that they exudate a resinous secretion, known as resin or colophony, which has several applications, including adhesives, soaps, and food additives (Beltran et al., 2016). The needles, leaves, and oils of plants belonging to the genus *Pinus* have been widely employed in folk medicine because of their numerous pharmacological properties, such as anti-aging and anti-inflammatory effects, and of the possibility of using them to treat liver diseases, skin diseases, and hypertension (Xie et al., 2015).

*Pinus elliottii* Engelm. and *Pinus tropicalis* Morelet are trees that can be as tall as 15–30 m, but which have been little studied as to their pharmacological properties. *P. elliottii* originates in southeastern United States, though it is widely cultivated as a subtropical crop in Brazil, India, and China. It is usually employed to fabricate oleoresin and furniture (Caetano da Silva et al., 2014), and its anthelmintic (Tóro et al., 2003) and antibacterial actions have been demonstrated (Caetano da Silva et al., 2014; Leandro et al., 2014). *P. tropicalis* is endemic to the island of Cuba and exhibits strong insecticidal (Leyva et al., 2009) and fungicidal (Andrade et al., 2014) effects.

Concerning resins obtained from *Pinus* species, phytochemical studies have shown that they consist mainly of diterpenes, which present countless known biological activities like anti-inflammatory, antifungal, antiparasite, and antibacterial actions (Caetano da Silva et al., 2014). DHA is an abietane diterpene that can be obtained from *Pinus* resin or commercial resin. It has already been described as the major compound in *P. elliotti* (Caetano da Silva et al., 2014). Researchers have investigated the DHA pharmacological potential and explored its ability to generate derivatives that can act as synthetic precursors of heteroaromatic compounds with promising biological properties. Indeed, gastroprotective, antiviral, antibacterial, and antiulcer activities have already been described for DHA or its derivative (Wada et al., 1985; Fonseca et al., 2004; Sepúlveda et al., 2005; Caetano da Silva et al., 2014; Leandro et al., 2014).

Considering the impact of dental caries on public health and the popular use and wide biological potential described for species belonging to the genus *Pinus*, here we investigate the antibacterial activity of *P. elliottii* and *P. tropicalis* resins and of the diterpene DHA, isolated from *P. elliottii*, against cariogenic bacteria. In addition, the lack of studies on the cytotoxicity of these resins has motivated us to address this issue along with the SI of the resins in relation to human cells.

## Materials and Methods

### Resin Acquisition and Tested Microorganisms

Authentic *P. elliottii* (batch number 21811-09) and *P. tropicalis* (batch number 20511-09) resin samples were obtained from the Brazilian Association of Resinators (ARESB), located in the city of Avaré – SP, Brazil. DHA was isolated from the *P. elliottii* resin and was obtained by the method previously described by Leandro et al. (2014) (Supplementary Figures S1, S2).

The following bacteria from the American Type Culture Collection (ATCC) collection were used to conduct the antibacterial assays: *S. mutans* (ATCC 25175), *S. mitis* (ATCC 49456), *S. sanguinis* (ATCC 10556), *S. sobrinus* (ATCC 33478), *S. salivarius* (ATCC 25975), *Lactobacillus casei* (ATCC 11578), and *Enterococcus faecalis* (ATCC 4082). These microorganisms were maintained in a freezer at −80°C in 20% glycerol solution in the Laboratory of Applied Microbiology Research (LaPeMA) – University of Franca (Unifran).

### Minimum Inhibitory Concentration and Minimum Bactericidal Concentration

The MIC is defined as the lowest concentration of the sample that can inhibit bacterial growth. MIC was determined by microdilution in microplates. The experiments were accomplished in 96-well microplates and were repeated three times.

Briefly, 1.0 mg/mL resin solutions were prepared in DMSO (Merck, Darmstadt, Germany) and subsequently diluted in BHI broth (Difco, Detroit, MI, United States). Then, the resin samples were tested at concentrations ranging from 0.195 to 400 μg/mL. The final DMSO content in the samples was 5% (v/v); five inoculated wells containing DMSO at concentrations ranging from 5 to 1% were used as negative controls. The inoculum was adjusted for each organism at 625 nm in a spectrophotometer, to give a cell concentration of 5 × 10^5^ CFU/mL, which corresponded to the final concentration in the well (CLSI, 2012). One inoculated well was included to control broth adequacy for organism growth. One non-inoculated well, free of antimicrobial agent, was also employed, to ensure medium sterility. The positive control was CHD (Sigma, St. Louis, MO, United States) at concentrations ranging from 0.115 to 59 μg/mL. Next, the microorganisms were incubated at 37°C for 24 h. *S. mutans*, *S. mitis*, *S. sanguinis*, *S. sobrinus*, and *L. casei* were incubated under microaerophilic conditions (atmosphere containing 10% CO_2_), while *S. salivarius* and *E. faecalis* were incubated under aerobic conditions.

After incubation, 30 μL of 0.02% resazurin (Sigma) aqueous solution was added to each well. Resazurin is an oxidation-reduction probe that allows microbial growth to be immediately detected. The blue and red colors represent absence and presence of microbial growth, respectively (Sarker et al., 2007).

To determine the MBC, a 10-μL aliquot of the inoculum was removed from each well before resazurin was added. The inoculum aliquot was then seeded on Blood Agar Base (Difco) supplemented with 5% defibrillation sheep blood for all strains. Next, the microplates were incubated as described above. MBC is defined as the lowest concentration of the sample where no bacterial growth occurs. A substance is considered to exert a bacteriostatic effect when its MBC value is higher than its MIC value. However, a substance is considered to exhibit a bactericidal effect when its MBC value is the same as its MIC value.

## Minimum Inhibitory Concentration Against the Biofilm

Preliminary tests were performed to define the best conditions for the biofilm to form, such as the ideal inoculum concentration and the period that is necessary for the biofilm to grow. For this purpose, the biofilms were standardized in three repeated experiments; BHI (Difco) broth and 96-well flat-bottom microplates were used. This assay was carried out for the bacteria that afforded the most promising MIC and MBC results. The best inoculum concentration and the optimal biofilm formation time were 10^6^ CFU/mL and 24 h, respectively (data not shown).

Minimum inhibitory concentration against the biofilm is defined as the minimum concentration of the *P. elliottii* or *P. tropicalis* resin that can inhibit biofilm formation by at least 50%. MICB_50_ was determined as described by Wei et al. (2006) with the modifications depicted below. Briefly, serial twofold resin dilutions were prepared in a 96-well polystyrene tissue culture plate (TPP, Trasadingen, Switzerland) containing BHI (Difco) broth for all the bacteria, as described previously. The final concentrations ranged from 0.195 to 400 μg/mL for the resins and between 0.115 and 59 μg/mL for the positive control (CHD; Sigma). Bacterial strains in the absence of antibacterial agent were employed as negative controls, and the inoculums were adjusted to give a cell concentration of 10^6^ CFU/mL for all the bacteria. The well contents were discarded after incubation at 37°C for 24 h. Then, each well was washed three times with 150 μL of sterile Milli Q water and fixed with 150 μL of methanol for 20 min. The whole experiment was repeated three times.

By following the procedure reported by Stepanović et al. (2007), the biofilms were quantified by OD by adding 150 μL of crystal violet (2%) to the microplate wells. After 15 min at room temperature, excess dye was removed by rinsing with tap water, followed by air-drying at room temperature. Then, 150 μL of glacial acetic acid 33% was gently added to each well, to re-solubilize the dye bound to the cells. The microtiter plate was covered with the lid and kept at room temperature for at least 30 min to minimize evaporation. The OD of each well was measured at 595 nm with the aid of a microtiter plate reader (ASYS, Eugendorf, Salzburg, Austria). The percentage of inhibition was calculated by using the equation (1 - At_595_/Ac_595_) × 100, where At_595__nm_ and Ac_595__nm_ are the absorbance of the wells treated with the resins and the control, respectively (Wei et al., 2006).

Cell viability under antibiofilm activity was measured by counting the number of microorganisms; the same procedures described above were employed in another microplate. The assay was conducted according to Moreti et al. (2017) with the adaptations depicted below. Dilutions between 10^−1^ to 10^−7^ were prepared with BHI (Difco) broth, to allow microorganism counting. Subsequently, 50 μL of each dilution was transferred to BHI (Difco) agar plates supplemented with 5% defibrillation sheep blood, divided into eight parts, and incubated under the conditions described previously. After incubation, the colonies were counted. Results are expressed as CFU/mL. In addition, the IC_50_ (concentration of resins capable of inhibiting 50% of microbial growth) was calculated by using GraphPad Prism 6, and results are expressed in μg/mL.

### Fractional Inhibitory Concentration Index (FICI)

The activities of combinations of one of the resins with CHD (Sigma) were evaluated by FICI for *S. mutans*, *S. mitis*, *S. sanguinis*, *S. sobrinus*, and *L. casei*, the bacteria that presented the lowest MIC values. Checkerboard assays were performed according to the protocol previously described by White et al. (1996) to investigate the *in vitro* antimicrobial efficacy of the combination of the *P. elliottii* (antimicrobial agent A) or *P. tropicalis* (antimicrobial agent B) resin with CHD (antimicrobial agent C; Sigma). The whole experiment was repeated three times, and concentrations of each compound were combined by using a standard MIC format.

**Table 1:** Minimum inhibitory concentration (MIC) and minimum bactericidal concentration (MBC) in μg/mL of the *P. elliottii* and *P. tropicalis* resins and of dehydroabietic acid (DHA) against bacteria that cause caries.

Bacterial strains	*P. elliottii* (μg/mL)		*P. tropicalis* (μg/mL)		DHA (μg/mL)		CHD (μg/mL)	
	MIC	MBC	MIC	MBC	MIC	MBC	MIC	MBC
*Streptococcus mutans* (ATCC 25175)	12.5	25	12.5	25	50	100	0.92	1.84
*Streptococcus mitis* (ATCC 49456)	25	25	12.5	12.5	25	25	3.68	3.68
*Streptococcus sanguinis* (ATCC 10556)	12.5	12.5	25	25	50	50	0.92	0.92
*Streptococcus sobrinus* (ATCC 33478)	25	25	25	25	100	100	0.46	0.92
*Streptococcus salivarius* (ATCC 25975)	100	100	50	100	100	100	0.92	0.92
*Lactobacillus casei* (ATCC 11578)	100	200	25	25	50	100	0.92	0.92
*Enterococcus faecalis* (ATCC 4082)	200	400	100	200	400	400	7.37	7.37

*DHA, dehydroabietic acid; CHD, chlorhexidine.*

Serial twofold dilutions of resins and CHD (Sigma) were mixed in each well of a 96-well microtiter plate. Fifty-microliter aliquots of the first and the second antimicrobial agents were added in vertical and horizontal orientation, respectively. A volume of 100 mL of fresh bacterial suspension (1 × 10^6^ CFU/mL) was added to each well and incubated as mentioned previously. FICIs were calculated according to the following formula: FICI = (MIC of antimicrobial agent A or B in combination / MIC of antimicrobial agent A or B alone) + (MIC of antimicrobial agent C in combination / MIC of antimicrobial agent C alone). FICI ≤ 0.5, >0.5–1.0, 1–4.0, and >4.0 was interpreted as synergistic, additive, indifferent, and antagonistic, respectively (Lewis et al., 2002).

### Cell Line and Culture Conditions and XTT-Based Cytotoxicity Assay

Human gingival fibroblasts (HGF cells) were employed to assess the cytotoxic potential of the *P. tropicalis* and *P. elliottii* resins. The cell line was maintained as monolayers in plastic culture flasks (25 cm^2^) in HAM-F10/DMEM (1:1, Sigma) culture medium supplemented with 10% fetal bovine serum (Nutricell), antibiotics (0.01 mg/mL streptomycin and 0.005 mg/mL penicillin; Sigma), and 2.38 mg/mL Hepes (Sigma), at 37°C with 5% CO_2_.

The cytotoxic activity was measured with the *in vitro* Toxicology Colorimetric Assay Kit (XTT; Roche Diagnostics) according to the manufacturer’s instructions. Extracellular XTT reduction occurs on the plasma membrane surface via transmembrane electron transport. This assay is widely used to determine cell proliferation or the cytotoxic effects of chemicals (Bernas and Dobrucki, 1999; Ferreira et al., 2016). For these experiments, the cells (10^4^ cells/well) were plated onto 96-well microplates. Each well received 100 μL of HAM F10/DMEM medium containing *P. tropicalis* or *P. elliottii*. The tested concentrations ranged from 1.2 to 2,500 μg/mL. The negative (without treatment), solvent (DMSO 1%; Sigma), and positive (DMSO 25%) controls were included. After incubation at 37°C for 24 h, the medium was removed; the cells were washed with 100 μL of PBS and exposed to 100 μL of HAM-F10 medium without phenol red. Then, 25 μL of XTT was added to each well. The microplates were covered and incubated at 37°C for 17 h. The samples had their absorbance determined by using a multiplate reader (ELISA – Asys – UVM 340/Microwin 2000) operating at a test wavelength of 450 nm and a reference wavelength of 620 nm. Cell viability is expressed as percentage of untreated cells, which served as the negative control group and was designated as 100%; hence, results are expressed as a percentage of the negative control. The antiproliferative activity was assessed by using the parameter of 50% inhibition of cell line growth (IC_50_). The experiments were repeated three times.

The SI of the samples was calculated by using the formula SI = IC_50_/MIC; the concentrations that reduced cell viability in HGF cells by 50% (IC_50_) and the MIC against the tested bacteria were taken into account (Elisha et al., 2017).

## Results

Table 1 lists the MIC and MBC values determined for the *P. elliottii* and *P. tropicalis* resins and for DHA against seven cariogenic bacteria. The values ranged from 12.5 to 400 μg/mL for the resins and from 25 to 400 for DHA. DMSO alone (5% concentration; negative control) had no effect on the tested bacteria.

The resins and DHA had the same MBC and MIC values against most of the tested bacteria, which meant that they exerted a bactericidal effect in these cases. The *P. elliottii* resin exhibited bacteriostatic action against *S. mutans*, *L. casei*, and *E*. *faecalis*, whereas the *P. tropicalis* resin showed this same effect on *S. mutans, S. salivarius*, and *E. faecalis*. DHA displayed bacteriostatic activity against *S. mutans* and *L. casei* (Table 1).

The *P. elliottii* resin presented promising results against *S. mutans*, *S. mitis*, *S. sanguinis*, and *S. sobrinus*, while *P. tropicalis* showed promising activity against all the investigated bacteria except *E. faecalis* (≥100 μg/mL; Table 1). DHA did not provide promising MIC results against any of the evaluated bacteria (≥10 μg/mL; Table 1).

The remaining experiments only involved the bacteria for which the resins yielded the best MIC results.

**Table 2:** Minimum inhibitory concentration against the biofilm and cell viability determined for the *P. elliottii* and *P. tropicalis* resins against cariogenic bacteria.

Bacterial strains	*P. elliotti*				*P. tropicalis*				CHD^a^			
	MICB_50_^b^		Cell viability^c^		MICB_50_		Cell viability		MICB_50_		Cell viability	
	μg/mL	%	Log_10_ (CFU/mL)	IC_50_ (μg/mL)	μg/mL	%	Log_10_ (CFU/mL)	IC_50_ (μg/mL)	μg/mL	%	Log_10_ (CFU/mL)	IC_50_ (μg/mL)
*Streptococcus mutans* (ATCC 25175)	100	50.78	5.95	1.95	6.25	50.90	5.94	2.53	0.23	85.33	0.20	1.37
*Streptococcus mitis* (ATCC 49456)	6.25	50.13	6.00	103.05	3.12	50.55	5.50	30.48	14.7	56.53	5.50	2.69
*Streptococcus sanguinis* (ATCC 10556)	200	53.59	4.00	6.67	^∗^	^∗^	^∗^	^∗^	1.84	76.79	7.01	2.63
*Streptococcus sobrinus* (ATCC 33478)	^∗^	^∗^	^∗^	^∗^	400	50.86	4.44	0.67	0.46	61.18	0.50	0.93
*Streptococcus salivarius* (ATCC 25975)	^∗^	^∗^	^∗^	^∗^	0.78	64.80	4.10	0.68	0.46	64.80	7.10	1.43
*Lactobacillus casei* (ATCC 11578)	^∗^	^∗^	^∗^	^∗^	3.12	50.72	5.50	5.45	0.92	58.37	1.19	1.06

*^a^chlorhexidine; ^b^MICB_50_ obtained by optical density, results are given in concentrations (μg/mL) and in percentage (%) of biofilm inhibition; ^c^cell viability obtained by count of microorganisms, results are given in Log_10_ CFU/mL and IC_50_ (calculated by graphpad prism 6); ^∗^: not rated.*

The MICB_50_ values ranged from 6.25 to 200 μg/mL for the *P. elliottii* resin against *S. mutans*, *S. mitis*, and *S. sanguinis*, and from 0.78 to 400 μg/ mL for the *P. tropicalis* resin against *S. mutans*, *S. mitis*, *S. sobrinus*, *S. salivarius*, and *L. casei*. The IC_50_ values for the microorganism count ranged from 1.95 to 103.05 μg/mL for the *P. elliottii* resin and from 0.68 to 30.48 μg/mL for the *P. tropicalis* resin (Table 2).

The FICI obtained for the resins combined with CHD (Sigma) revealed that the combination between the *P. elliottii* resin and CHD (Sigma) exerted an antagonistic effect on *S. mutans* and *S. sanguinis* and an indifferent effect on *S. mitis*. The combination between the *P. tropicalis* resin and CHD (Sigma) had an indifferent effect on the three bacteria (Table 3).

**Table 3:** Fraction Inhibitory Concentration Index (FICI) of the *P. elliottii* (PE) or the *P. tropicalis* (PT) resin in combination with chlorhexidine (CHD) against cariogenic bacteria.

Bacterial strains	MIC PE	MIC CHD	FIC PE	FIC CHD	FICI	Interpretation	MIC PT	MIC CHD	FIC PT	FIC CHD	FICI	Interpretation
*Streptococcus mutans* **(**ATCC 25175)	12.5	0.46	50	0.46	5	Antagonistic	12.5	0.46	25	0.23	2.5	Indifferent
*Streptococcus mitis* **(**ATCC 49456)	25	1.84	50	3.68	4	Indifferent	25	1.84	25	1.84	2	Indifferent
*Streptococcus sanguinis* **(**ATCC 10556)	12.5	0.92	12.5	3.68	5	Antagonistic	25	0.92	25	0.92	2	Indifferent

**FIGURE 1:**
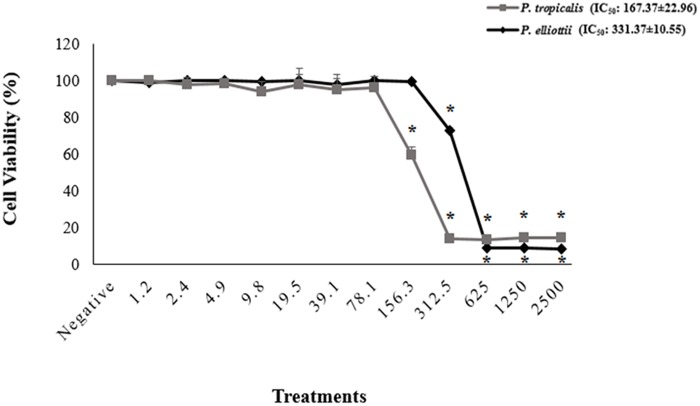
Cell viability (%) of HGF cells after treatment with the *P. tropicalis* and the *P. elliottii* extracts. Negative control (no treatment); the solvent (DMSO 1%) group showed cell viability of 99.84%; the positive control (DMSO 25%) showed cell viability of 9.27%. Values are means ± standard deviations. ^∗^Significantly different from the negative group (*P* < 0.05).

Figure 1 represents the mean of three independent cell viability experiments, and the bars indicate the standard error of the mean. Cell viability reduced significantly at *P. elliottii* and *P. tropicalis* resin concentrations above and equal to 312.5 μg/mL (IC_50_ 331.37 ± 10.55 μg/mL) and 156.3 μg/mL (IC_50_ 167.37 ± 22.96), respectively, indicating that the resins at these concentrations had a cytotoxic effect on the cells. Except for *P. tropicalis* for *E. faecalis*, SI demonstrated that both resins were at least once more selective toward bacterial cells than toward normal human cells (Table 4).

## Discussion

Biofilms can naturally emerge in dental enamel, but poor oral hygiene can lead to biofilm accumulation, which is not compatible with an individual’s health (Marsh, 1992). With this concern in mind, we decided to investigate the antibacterial activity of two resins of the genus *Pinus* and of an isolated compound, the diterpene DHA, against cariogenic bacteria in the sessile and in the planktonic mode.

According to Ríos and Recio (2005) and Gibbons (2008), plant extracts (as well as essential oils) and isolated compounds with demonstrated activity at concentrations below 100 and 10 μg/mL, respectively, have promising biological action. Here, the *P. elliottii* and *P. tropicalis* resins proved to be promising against most of the tested bacteria, while DHA did not yield satisfactory MIC results. In fact, the literature asserts that plant resins and extracts often exhibit more pronounced biological activities than their isolated constituents. This is usually due to synergistic, additive, or potentiating interactions between the constituents, which generally involve action on multiple targets, resulting in significant biological potential (Raskin et al., 2002; Schmidt et al., 2008).

We did not achieve any promising results against *E. faecalis* for any of the tested resins or DHA. This could be due to the high levels of resistance that have already been described for this microorganism. Barbosa-Ribeiro et al. (2016) reported that, because of their morphologic and genetic characteristics, *E. faecalis* might be resistant to intracanal procedures and even to systemic antibiotics. The *E. faecalis* resistance mechanisms result from physiological or structural changes in the bacterial cell, which is a survival strategy against attack by antimicrobial agents (Barbosa-Ribeiro et al., 2016). Our results corroborate with the data published by Leandro et al. (2014), who used multiresistant strains of this microorganism and obtained MIC values of 100 and 50 μg/mL against *E. faecalis* (NCTC 14990) and 100 and 25 μg/mL against *E. faecalis* (clinical isolate) for the *P. elliottii* resin and DHA, respectively. On the other hand, in the same study, the authors found promising results against *Staphylococcus epidermidis* (ATCC 14990): MIC values were 25 μg/mL and 6.25 μg/mL for the *P. elliottii* resin and DHA, respectively.

**Table 4:** Determination of the SI of *P. tropicalis* and *P. elliottii* resins.

Bacterial strains	Selectivity index (IC_50_/MIC)	
	*P. tropicalis*	*P. elliotii*
*Streptococcus mutans*	13.39	26.51
*Streptococcus mitis*	6.69	13.25
*Streptococcus sanguinis*	13.39	26.51
*Streptococcus sobrinus*	6.69	13.25
*Streptococcus salivarius*	1.67	3.31
*Lactobacillus casei*	1.67	3.31
*Enterococcus faecalis*	0.84	1.65

*IC_50_, inhibitory concentration of 50% of cell viability; MIC, minimum inhibitory concentration.*

Caetano da Silva et al. (2014) evaluated the antibacterial potential of the *P. elliottii* resin and DHA against endodontic anaerobic bacteria. As in the case of our study, the resin afforded promising MIC values, which ranged from 0.4 to 50 μg/mL. In contrast, DHA only showed promising antibacterial activity against *P. gingivalis* (ATCC 33277), with MIC of 6.2 μg/mL. Together with our results, the results of Caetano da Silva et al. (2014) revealed that the *P. elliottii* resin is promising against different microorganisms, whereas DHA, despite its already demonstrated antimicrobial potential, seems to rely on a synergistic action among the resin constituents to act against cariogenic bacteria.

The antibacterial activity of the genus *Pinus* has been explored over the years (Kim and Shin, 2005; Feng et al., 2010; Merah et al., 2018). However, the antibacterial potential of *P. tropicalis* has not been assessed yet, so we had difficulty comparing our data with literature results. Nevertheless, the present study will aid later works that address this issue and is the first study that has verified that the *P. tropicalis* resins exhibit significant antibacterial activity against cariogenic bacteria.

Stoddart (2011) reported that it is essential to conduct other assays when we evaluate the efficacy of a possible antimicrobial agent. For this reason, we also assessed the antibacterial potential of the *Pinus* resins by other techniques. We determined MICB_50_ to examine whether the *P. elliotti* and *P. tropicalis* resins can prevent the target bacteria from forming biofilm. We performed this assay, as well as FICI experiments with the bacteria for which the resins gave the best MIC results.

The resins tested herein provided promising MICB_50_ values against the studied bacteria as demonstrated by both OD reading and IC_50_ given by microorganism counts. Our results were even better than the results obtained by MIC. For some bacteria (*S. mitis* for both resins and *S. salivarius* for the *P. tropicalis* resin), the results were even better than the results achieved with the positive control, CHD (Sigma). Biofilms respond differently from planktonic cells depending on the growth phase, period of exposure to the antibacterial agent, and antimicrobial concentration (Gursoy et al., 2009). In other words, the same bacterium displays distinct behavior when it is in the biofilm mode and in the planktonic state, a fact that must be considered during all laboratory tests (Signoretto et al., 2012). Wei et al. (2006) highlighted that preventing biofilm formation is extremely relevant because inhibiting biofilm growth also avoids its maturing, which underscores the importance of the results obtained in our study.

This is the first work that has evaluated the MICB_50_ of species belonging to the genus *Pinus*. However, assessment of the antibiofilm activity of DHA (extracted from the *P. elliottii* resin) against endodontic bacteria revealed significant results, and MICB_50_ lay between 7.8 and 62.5 μg/mL and prevented all the bacteria from forming a biofilm. For some bacteria, the results were lower as compared to the values determined for the reference drug, CHD (Sigma).

It is unusual for bacterial inhibition values of bacteria in the sessile mode to be lower than or equal to bacterial inhibition values of bacteria in the planktonic mode because biofilms are considered to be more resistant to antimicrobial agents as compared to planktonic cells (Stewart and Costerton, 2001). However, this is not unprecedented in the literature (Al-Shuneigat et al., 2005; Karpanen et al., 2008; Fernández et al., 2018). According to Karpanen et al. (2008), unexpected results such as this suggest that the extracellular matrix of the bacterial biofilm may alter the activity of the tested substances, or that substances consisting of several heterogeneous, hydrophilic, and hydrophobic properties may alter ionic interactions in the biofilm extracellular matrix and act on the same target on the bacterial cell. Therefore, further studies are necessary to establish the modes of action of the *Pinus* resins investigated herein. We emphasize that spectrophotometric readings (OD) and microorganism count (log_10_ CFU/mL) are recommended to show the ability of antimicrobial agents to inhibit biofilm formation (antibiofilm activity). Together, the techniques applied here can provide reliable results concerning biofilm activity (Kharazmi et al., 1999; Pettit et al., 2005; Caetano da Silva et al., 2014).

The literature has recorded the increased resistance of microorganisms to antiseptics, such as CHD, in addition to their potential cytotoxicity to normal cells (Faria et al., 2007; Lessa et al., 2010; Cieplik et al., 2018). Therefore, the MICB_50_ values found here are interesting, demonstrating that these *Pinus* resins have potential action against biofilms formed by microorganisms that participate in the formation of dental caries. In other words, these resins deserve to be explored since they may represent an alternative to the antibacterial chemical agents that have been traditionally employed.

Regarding cytotoxicity, all the promising concentrations of the resins were below the cytotoxic concentrations. The SI demonstrated that both resins are more selective toward bacterial cells than toward normal human cells for most of the tested bacteria. This selectivity is even more evident if we consider the activity of the *P. elliottii* resin against *S. mutans* and *S. sanguinis* and the action of *P. tropicalis* against *S. mutans*, *S. mitis*, *S. sobrinus*, and *S. sanguinis –* in these cases, selectivity toward bacterial cells is at least ten times higher than selectivity toward normal human cells. This is important because it indicates that the resins can be used at concentrations up to 10-fold or higher than their MIC without being cytotoxic to normal cells.

Combinations of one of the resins with CHD (Sigma) do not exhibit any additive effects. A search of the scientific literature did not retrieve any articles on the synergism of *P. elliottii*, *P. tropicalis*, or other *Pinus* species, which made comparison of our results with data by other authors impossible. Despite the absence of synergistic effects when the resins are combined with CHD (Sigma), the *P. elliottii* and *P. tropicalis* resins showed promising results during the other antimicrobial tests performed herein. In this sense, this is the first study that has revealed that an association of the resins tested here with CHD (Sigma) is not recommended. On the other hand, the use of natural products may be an economical approach to prevent and to treat dental caries (Wassel and Khattab, 2017). Considering that phytotherapy constitutes a form of medicinal therapy that has notably grown in recent years (Silva et al., 2015), additional research involving the use of *P. elliottii* and *P. tropicalis* as possible phytotherapics against cariogenic bacteria is encouraged.

## Conclusion

The results of the present study do not demonstrate any activity of DHA against the tested bacteria. On the other hand, our data reveal the *in vitro* antibacterial activity of the *P. elliottii* and *P. tropicalis* resins against cariogenic bacteria in the planktonic and in the sessile modes. The promising MIC/MBC values are lower than the resin concentrations that provide 50% viability for human gingival fibroblast cells. Additionally, on the basis of the SI, the resins are more selective toward the bacterial cells than toward the normal cells. As for the evaluation of the combinations of one of the resins with CHD, results point to antagonism or indifference. Judging from the values of MIC of biofilm obtained herein, the resins can inhibit all the assayed bacteria by at least 50%. These results encourage further studies to unveil the action mechanisms of these resins and contribute to the quest for new effective and less toxic alternatives that can inhibit bacterial growth and reduce the incidence of dental caries.

## Author Contributions

CM, DT, RV, and SA conceived the idea for this study. KS, JD, FA, and CM participated in the study design, organized the data, and evaluated their quality. MOI and KS conducted the antibacterial assays. NF conducted the cytotoxicity assay. CM, RV, SA, and DT critically reviewed the manuscript. All authors have read and approved the final manuscript.

## Conflict of Interest Statement

The authors declare that the research was conducted in the absence of any commercial or financial relationships that could be construed as a potential conflict of interest.

## Abbreviations

BHI: brain heart infusion
CHD: chlorhexidine
DHA: dehydroabietic acid
DMSO: dimethylsulfoxide
FICI: fractional inhibitory concentration index
HGF: human gingival fibroblasts
IC_50_: 50% inhibitory concentration
MBC: minimum bactericidal concentration
MIC: minimum inhibitory concentration
MICB_50_: minimum inhibitory concentration against the biofilm
OD: optical density
PBS: phosphate buffered saline
SI: selectivity index.
