# Supplementary figures and images for: Antibacterial and Cytotoxic Activities of Pinus tropicalis and Pinus elliottii Resins and of the Diterpene Dehydroabietic Acid Against Bacteria That Cause Dental Caries
Source: Front Microbiol. 2019 May 7;10:987. doi: 10.3389/fmicb.2019.00987 (PMC6514049; doi:10.3389/fmicb.2019.00987)

SpinWorks 4: no title

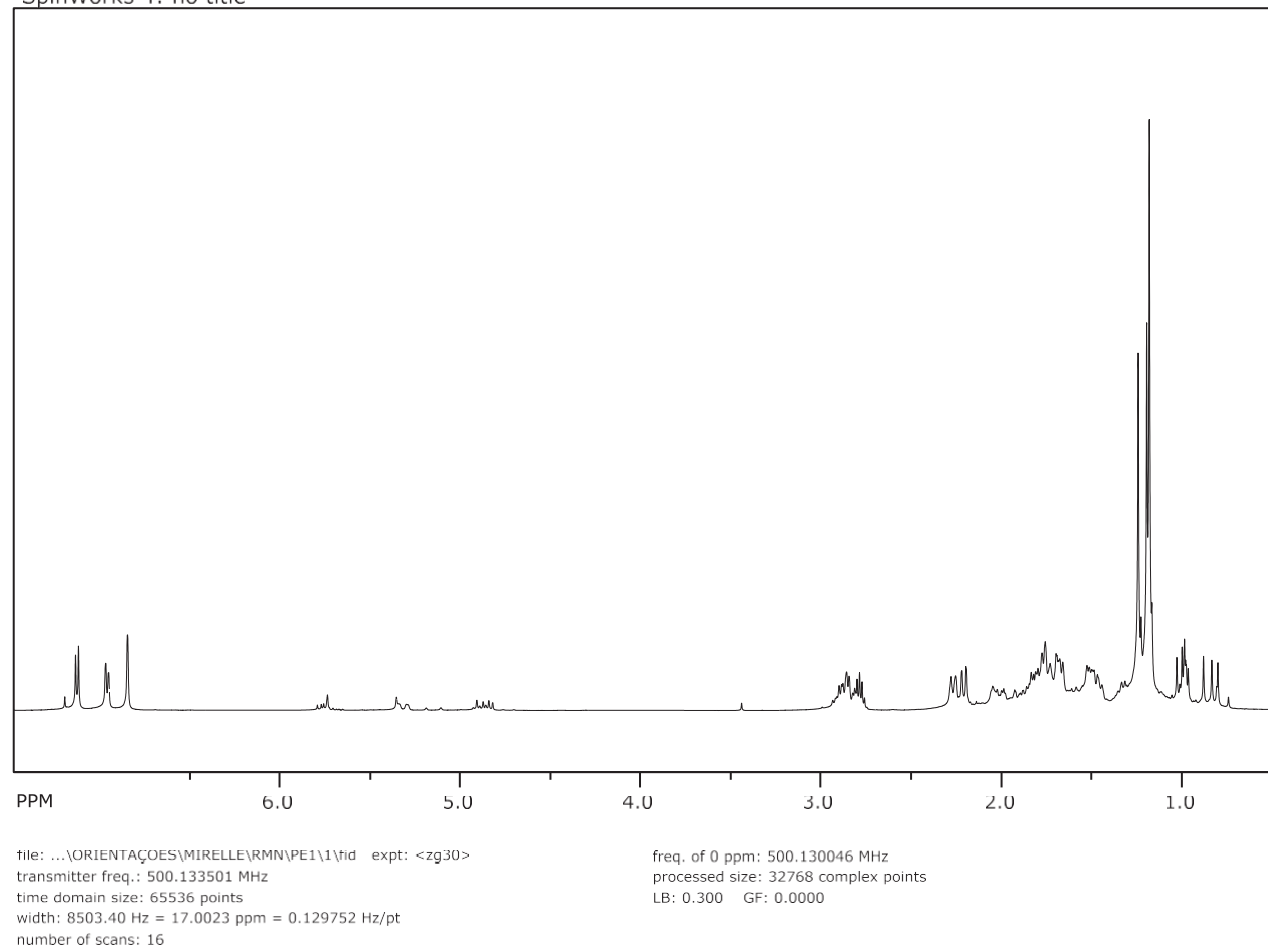

Figure S1.  $^1\text{H}$ -NMR Spectrum of Dehydroabietic acid (DHA).  $\text{CDCl}_3$  / 500 MHz

Supplement: Supplementary file 1 [file Data_Sheet_1.pdf]

SpinWorks 4: no title

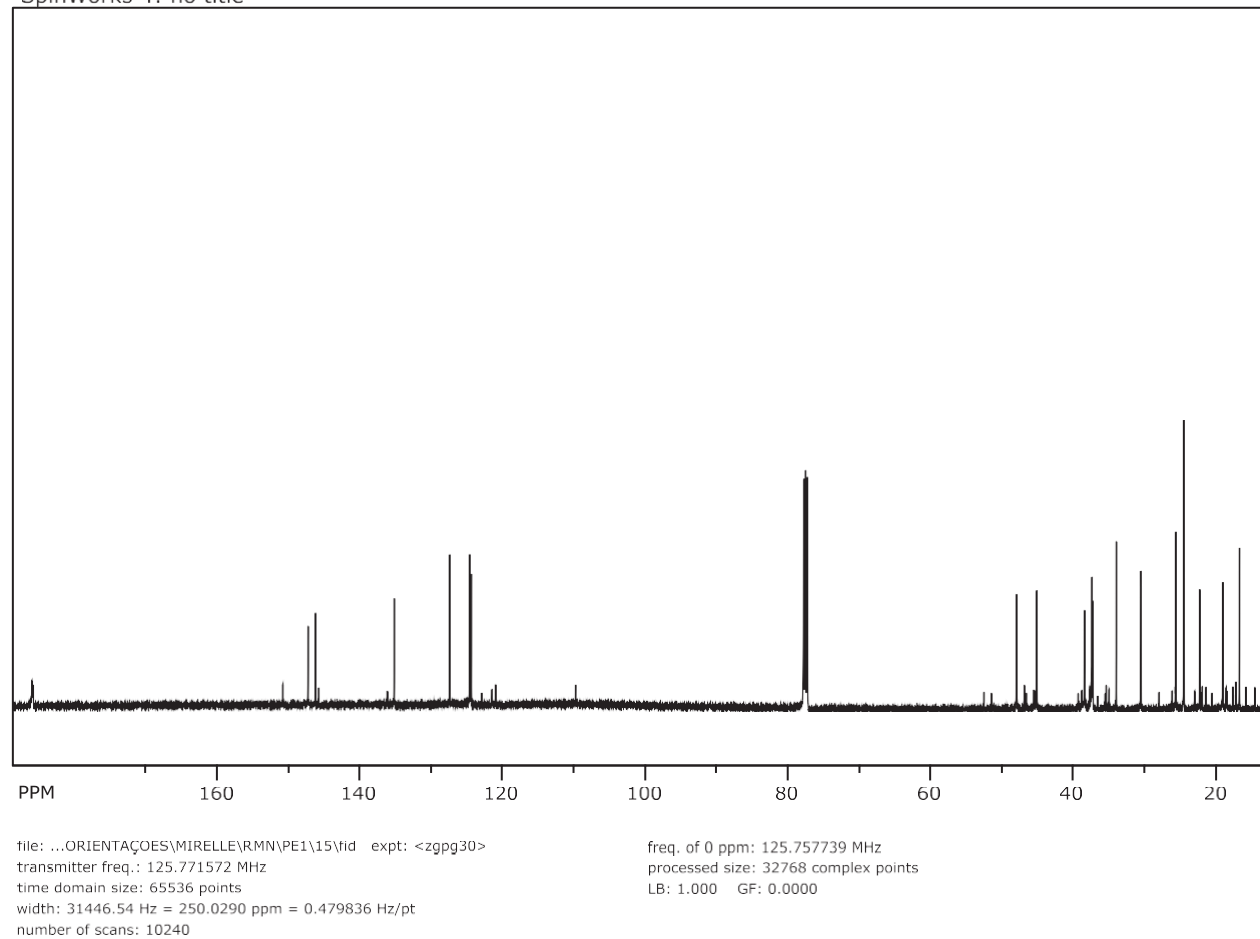

Figure S2.  $^{13}\text{C}$ -NMR Spectrum of Dehydroabietic acid (DHA).  $\text{CDCl}_3$  / 125 MHz

Supplement: Supplementary file 2 [file Data_Sheet_2.pdf]
